# *Borrelia burgdorferi*—A Bacterium Worthy of Consideration in Culture-Negative Prosthetic Joint Infection

**DOI:** 10.5435/JAAOSGlobal-D-23-00068

**Published:** 2023-09-14

**Authors:** Mary Crowe, Mario Giacobazzi, Edward Griffin, Shawn Storm

**Affiliations:** From the Lake Erie College of Osteopathic Medicine, Erie, PA.

## Abstract

A 68-year-old woman presented to the orthopaedic office with 2 weeks of atraumatic right prosthetic knee pain and swelling. She previously lived pain free and fully functional after a total knee arthroplasty 8 years ago. Initial radiographs showed a small joint effusion, and serum inflammatory markers were elevated. Arthrocentesis yielded 12ccs of culture-negative cloudy serous fluid containing 3,270 white blood cells, 92% polymorphonuclear neutrophils. The patient underwent prosthesis explant, antibiotic spacer placement, and began empiric IV antibiotic therapy as stage one of a planned two-stage revision. Intraoperative tissue cultures were negative, and the postoperative plan was to continue IV vancomycin for a total of 6 weeks. Two weeks post-op, serum Lyme antibody testing returned positive. The patient was switched to doxycycline and ceftriaxone for a total duration of 4 weeks, followed by a successful second-stage revision and remains asymptomatic after 1 year. Five cases of culture-negative prosthetic joint infections caused by the spirochete, *Borrelia burgdorferi*, have been reported in the orthopaedic literature.^1-4^ We present a sixth case, occurring in a 68-year-old woman in Northwestern Pennsylvania, 8 years after a primary right total knee arthroplasty.

## Case

Patient presented to the orthopaedic clinic with 2 weeks of right knee pain and swelling. She had undergone a right total knee arthroplasty at an outside hospital 8 years earlier and reported no previous episodes of pain, swelling, or mechanical symptoms regarding the knee. She denied any known trauma, fevers, body aches, or flu-like symptoms. One month before, she had been seen in the orthopaedic clinic for right hip pain. Then, she was diagnosed with trochanteric bursitis, which resolved after a greater trochanteric bursa injection of steroid.^1,2,3,4^

In the preceding 4 months, the patient had consulted her primary care provider because of complaints of fatigue. However, apart from that, she denied experiencing any other recent medical changes. The patient's medical history included type II diabetes managed with oral medications, hypertension, gastroesophageal reflux disease, hypothyroidism, depression, hyperlipidemia, and a 40 pack-year smoking history. The patient had no history of septic arthritis, malignancy, inflammatory disease, coagulopathy, anticoagulation therapy, and renal or liver failure. She denied any fevers or rashes in the last few months. Patient had a dog and lived in a rural setting. She denied any known tick bites in the past year or before the tick-borne illness.

On arrival to the orthopaedic office, the patient was nontoxic in appearance. She was afebrile with a temperature of 98° Fahrenheit, blood pressure 138/86, pulse rate 81 beats per minute, and body mass index of 37. On examination, there was a small right knee effusion, mildly increased warmth, and no notable erythema. Moderate medial and lateral joint line tenderness to palpation was noted. Her passive range of motion measured 0 to 100° with pain on terminal flexion. Varus and valgus stress testing in extension and flexion demonstrated no evidence of instability.

Work-up included four view radiographs of the knee, serum complete blood count (CBC) w/diff, erythrocyte sedimentation rate (ESR), and C-reactive protein (CRP). Radiographs showed no evidence of component loosening, periosteal reaction, osteolysis, generalized bone resorption, or subsidence. Inflammatory laboratory test results indicated an elevated ESR of 78 mm/ESR of 78mm/hour and a CRP level 34 mg/L. The serum white blood cell (WBC) count was 7.5. The patient was subsequently asked to return to the office for a joint aspiration procedure.

Arthrocentesis yielded 12cc of cloudy serous fluid with a cell count of 3,270 WBCs, of which 92% were neutrophils. Given the elevated serum inflammatory markers, cell count in the aspirate, and percentage of neutrophils, a Musculoskeletal Infection Society^5^ (MSIS) score of 8 was calculated (with a score >6 considered indicative of an infected). Based on this, the decision was made to admit the patient to the hospital and proceed with a two-stage revision. Initial gram stain of the clinic aspirate was negative for organisms with many polymorphonuclear neutrophils. The cultures from the aspirate showed no growth at the 72-hour mark and were held for an additional 3 weeks continuing to test negative for fastidious organisms. The patient underwent a standard prosthesis explant procedure, followed by the placement of an articulating antibiotic spacer. Both blood cultures and intraoperative tissue cultures were negative for growth. Postoperatively, a peripherally inserted central catheter line was established. An infectious disease (ID) specialist was consulted via Telehealth on postoperative day 1. and they recommended a 6-week course of IV vancomycin and ceftriaxone unless cultures returned positive. Arranging for home health services to administer daily IV infusions proved too costly for the patient, and she was not open to the option of skilled nursing care. Therefore, plans were made for the patient to receive daily infusions at the hospital instead. On postoperative day 5, the patient had an outpatient appointment with the ID physician. Given her residence in a rural setting and frequent walks with her dogs in wooded settings, the ID physician decided to conduct serum Lyme disease testing. The patient denied any known tick bites in the previous 2 years.

After 2 weeks of empiric antibiotic therapy, the patient’s serum Lyme test returned positive. The Lyme antibody screen returned elevated results of 5.06 (negative <0.90, 0.90 to 1.09 equivocal, >1.09 positive). The Lyme disease IgG Immunoblot indicated the presence of 8 of 10 specific borrelial proteins, marking a positive result. In addition, IgM reactivity was positive for one of the three specific borrelial proteins. She was switched to doxycycline 100 mg twice daily by mouth. After 2 weeks on doxycycline, she developed upset stomach and intolerance to the medication and was switched to 2 weeks IV ceftriaxone 2 g every 24 hours. Laboratory tests including ESR and CRP were trended. After completion of the antibiotics, the patient was given a 2 weeks holiday, after which joint aspiration was performed and sent for synovial Lyme PCR, and serum inflammatory markers were repeated. The postantibiotic holiday CRP level was 6.8 mg/L (normal <8.0) before the second-stage revision. The synovial Lyme disease PCR test was negative. Subsequently, the patient proceeded with the final second-stage revision surgery. The postoperative course was uneventful, and after a year, the patient exhibited no signs or symptoms of infection.

The patient discussed in this case report was informed that data concerning the case would be submitted for publication, and the patient agreed.

## Discussion

Identifying the causative microorganism in prosthetic joint infections (PJIs) is essential for efficient eradication of the organism, successful second-stage revision, and reduction in the total treatment expense and consequences of broad-spectrum antibiotic treatment.^6^ Negative culture results increase the degree of difficulty when treating a PJI. Between 2016 and 2021, five cases^1,2,3,4^ of culture-negative PJIs caused by the spirochete *Borrelia burgdorferi* were reported in the orthopaedic literature. We present a sixth case, occurring in a 68-year-old woman in Northwestern Pennsylvania, 8 years after a primary right total knee arthroplasty. Similar to the five previous reported cases, our patient presented with a painful, swollen joint, elevated serum inflammatory markers, and culture-negative aspirate with a high percentage of neutrophils. Aerobic and anerobic cultures remained negative. The patient's MSIS score was 8 (>6 considered infected).^5^ One week after the first-stage explant, *B. burgdorferi* was determined to be the causative microorganism. A second-stage revision was successfully performed after 6 weeks of antibiotic therapy. The patient remained pain-free and asymptomatic 1 year later.

The process of defining a culture-negative PJI remains difficult. The working consensus of the MSIS^5^ provides guidelines that assists surgeons in their diagnosis. The benchmark in the United States for a chronic PJI remains a two-staged revision.^7^ In this case, a two-staged revision was performed, although the treatment course was based on a suspected culture-negative PJI caused by one of the more common offending bacteria. The treatment of Lyme disease is based on experience in treating native septic knee arthritis caused by *B. burgdorferi*.^8^ The presence of orthopaedic hardware distinguishes the difference in the management of PJIs compared with native joint infections.

Orthopaedic implants are an ideal surface for the formation of the microorganism-embedded glycocalyx known as a biofilm.^9^ Bacterial biofilms create a complex, protective layer that renders bacteria highly resistance to antibiotic therapies and surgical irrigation and débridement with the retention of hardware.^7^ Sapi et al^10^ found substantial evidence that *B. burgdorferi* produces a biofilm in vitro, and this has historically been theorized to contribute to antibiotic resistance, recurrence, and chronic manifestations of Lyme disease. In a subsequent study, Sapi et al^11^ found and characterized in vivo production of biofilm by *Borrelia*. The orthopaedic surgeon should take this into careful consideration when deciding how to proceed in the management of PJI caused by *B. burgdorferi*.

Owing to the limited cases available in the academic literature, it can only be speculated as to whether medical therapy alone with antibiotics is adequate or if surgical intervention is required. The recent findings that *Borrelia* does in fact produce a biofilm that has been isolated in human tissue^11^ raises question as to whether PJI caused by *B. burgdorferi* should follow similar treatment algorithms as the more commonly encountered bacterial offenders. These authors agree that too little is known about Lyme PJI to make treatment recommendations and that the best treatment is theoretical at this time. Nonetheless, *B. burgdorferi* as a cause for culture-negative PJIs should be a consideration for those patients in endemic areas, with or without known tick exposure.
